# Severity of illness and risk of mortality in Mayo Clinic’s virtual hybrid advanced care at home program: a retrospective cohort study

**DOI:** 10.1186/s12913-023-09333-7

**Published:** 2023-03-27

**Authors:** Margaret R. Paulson, Ricardo A. Torres-Guzman, Francisco R. Avila, Karla C. Maita, John P. Garcia, Antonio J. Forte, Gautam V. Matcha, Ricardo J. Pagan, Michael J. Maniaci

**Affiliations:** 1 Division of Hospital Internal Medicine, Mayo Clinic Health Systems, Eau Claire, WI USA; 2 Division of Plastic Surgery, Mayo Clinic, Jacksonville, FL USA; 3 Division of Hospital Internal Medicine, Mayo Clinic, 4500 San Pablo Rd, Jacksonville, FL 32224 USA

**Keywords:** Home hospital, Telemedicine, Risk of mortality, Severity of illness

## Abstract

**Background:**

In July 2020, Mayo Clinic launched Advanced Care at Home (ACH), a high-acuity virtual hybrid hospital-at-home model (HaH) of care at Mayo Clinic Florida and Northwest Wisconsin, an urban destination medical center and a rural community practice respectively. This study aims to describe demographic characteristics of ACH patients as well as their acuity of illness using severity of illness (SOI) and risk of mortality (ROM), to illustrate the complexity of patients in the program, taking into account the different diagnostic related groups.

**Methods:**

Mayo Clinic uses All Patient Refined-Diagnosis Related Groups (APR-DRG) to calculate SOI and ROM on hospitalized patients. APR-DRG data, including SOI and ROM, were gathered from individual chart reviews from July 6, 2020, to March 31, 2022.

**Results:**

Out of 923 patients discharged from ACH, the average APR-DRG SOI was 2.89 (SD 0.81) and ROM was 2.73. (SD 0.92). Mean age was 70.88 (SD 14.46) years, 54.6% were male patients and the average length of stay was 4.10 days. The most frequent diagnosis was COVID-19 infection with 162 patients (17.6%), followed by heart failure exacerbation (12.7%) and septicemia (10.9%). The 30-day readmission rate after discharge from ACH was 11.2% (*n* = 103) and the 30-day mortality rate was 1.8% (*n* = 17). There were no in-program patient deaths.

**Conclusions:**

SOI and ROM from patients at the ACH program have been shown to be in the range of “moderate/major” according to the APR-DRG classification. The ACH program is capable of accepting and managing highly complex patients that require advanced therapeutic means. Furthermore, the ACH program has an in-program mortality rate of 0 to date. Therefore, ACH is rising as a capable alternative to the brick-and-mortar hospital.

**Supplementary Information:**

The online version contains supplementary material available at 10.1186/s12913-023-09333-7.

## Introduction

Delivering high-acuity hospital level care at home has increased steadily over the last decade [1]. This effort, often referred to as hospital-at-home (HaH) has the potential to solve for several current challenges in health care, including Emergency Department (ED) overcrowding and the high cost of medical care [1–4]. Past HaH programs have shown to be successful in treating patient with both moderate-acuity medical illnesses like acute bacterial pneumonia as well as complex clinical scenarios like congestive heart failure and neuromuscular disease [5, 6]. But as HaH expands as an alternate setting for inpatient-level care, the question of what level of inpatient acuity can be safely treated in this model remains.

The All Patient Refined-Diagnosis Related Groups (APR-DRG) system is used to measure hospitalized patient disease burden and has two subclasses, Severity of Illness (SOI) and Risk of Mortality (ROM) that are used by many United States (US) hospital systems [7]. APR-DRG metrics is a scale that ranges from 1 to 4, with 1 equaling “Low”, 2 equaling “Moderate”, 3 equaling “High” and 4 equaling “Extreme.” The ROM is the likelihood of mortality in the program [8]. The SOI is defined as the extent of physiologic decompensation or organ system loss of function and is designed to predict the number of medical resources needed to attend to an individual patient [8]. Hence, a more severe patient will require more resources and have a higher SOI score. The metrics are based on primary and secondary diagnoses, age and the procedures performed [9]. Both SOI and ROM are independent subclasses from each other, meaning that is possible to obtain a high SOI score with a low ROM [10]. Moreover, the Severity of Illness and Risk of Mortality metrics have shown to be better predictors than other classifications, such as Charlson comorbidity index (CCI) of in-hospital mortality among surgical patients [8]. APR-DRG is generated in a single algorithm which ensures a uniform methodology, unlike CCI that has multiple methods reported in the literature to classify the comorbidities [8]. Additionally, it is likely that APR-DRG will continue to be accurate in future years due to the annual update on new diagnostic and procedural codes.

Advanced Care at Home (ACH) is the Mayo Clinic virtual hybrid HaH program which began treating patients in 2020. This program treats multiple Mayo Clinic patients across the US, with medical care planning directed by providers in a command center located in Jacksonville Florida and executed through in-home services delivered by local medical suppliers [11]. This model was built with the intention of treating high-acuity, clinically stable hospital inpatients in the comfort of their own homes. The purpose of this study is to report the SOI and ROM APR-DRG scores of patients treated in ACH. We consider this study to be novel because, to date, there have been no previous studies in the literature that describe the APR-DRG SOI and ROM subclass classification from patients managed in HaH programs. We intend to show that patients with a higher ROI and SOI and, thus, a greater complexity are eligible to receive safe and effective management with the ACH model.

## Methods

### Patient selection and setting

This study was approved by the Mayo Clinic Institutional Review Board as a retrospective chart review under protocol number 20–010753 and analyzed the de-identified patient data under protocol number 21–004666. The study was conducted between July 6, 2020, and March 31, 2022, at three Mayo Clinic Hospitals: Mayo Clinic in Florida, a 304-bed community academic hospital in Jacksonville, Florida, Mayo Clinic Healths Systems Eau Claire, a 304-bed community hospital in Eau Claire, Wisconsin, and Mayo Clinic in Arizona, a 268-bed community academic hospital in Phoenix, Arizona. The inclusion criteria for this study were the following: 1) all patients admitted to the ACH program in Florida, Wisconsin, and Arizona, with no age restrictions. Patients were excluded from the study if they did if they had missing or unknown data. Admission to the ACH program is completely voluntary. Patients provide both oral and written consent to participate in the ACH program and all subsequent research and experience studies.

### ACH model of care

The ACH model of care has been described in detail previously [11, 12]. Briefly, ACH is a virtual hybrid model where patients receive inpatient care in the comfort of their own homes. Patients were admitted to ACH either directly from the emergency department directly to home, bypassing the hospital inpatient wards, or from the brick-and-mortar (BAM) hospital wards when clinically stable. Patients were screened prior to admission to the program to ensure clinical stability for the home setting and continued inpatient needs (Additional file 1: Appendix A). A social screening ensured that the home setting was safe for high-acuity care delivery for both the patient and the medical staff.

Patients were monitored from the comfort of their homes using a technology stack and a specially configured audio/video communication device to communicate with their clinical teams. Patients received in home care from advanced practice provider visits, rapid response services, phlebotomy, nursing care, meals, and diagnostic images such as abdominal and chest radiographs as they would have received in the BAM hospital setting. All ACH patients are seen twice daily by either registered nurse or a community paramedic overseen by the command center virtual registered nurse. Laboratory studies and basic radiographic exams, such as x-rays and ultrasound, are performed in the home. Intravenous medications are administered by a visiting nurse or community paramedic. Respiratory and physical therapy services can be done in the home and the medical plan overseen and adjusted by the virtual care team. When the patients reached a stable endpoint, they were discharged from the program with regular primary care provider follow-up.

### Data collection and statistical analysis

We used APR-DRG to calculate the severity of illness (SOI) and risk of mortality (ROM) among patients enrolled in ACH. With the help of APR-DRG, metrics regarding SOI and ROM were calculated using EPIC Healthy Planet (Verona, WI) and Optum (Optum One) to extract the data. Primary diagnostic related groups (DRG) were identified and divided into several categories such as: cardiology, gastroenterology, neurology, pulmonary, among others. Data collection was extracted from medical charts, administrative record abstractions, and insurance claims to document diagnoses. In addition, demographic characteristics including patient age, sex, and average length of stay (ALOS) were collected. Thirty-day readmission and mortality rates were collected; the 30-day period begins from the point of discharge from ACH. If a patient care escalation requiring return to the brick-and-mortar hospital occurred while the patient was enrolled in ACH, this did not count as a readmission as it was still part of the initial inpatient encounter. In-program mortality rate was also collected.

After data extraction from the electronic medical record regarding demographic characteristics and classification of each patient into one of the diagnostic related groups, an average and standard deviation (SD) measurements were performed of the independent variables of age, SOI and ROM using SPSS Statistics (IBM, NY). The SOI and ROM of the COVID-19 and non-COVID-19 patients were calculated and compared using the unpaired Mann–Whitney U-test with a *p*-value of < 0.05 being considered statistically significant.

## Results

A total of 934 patients were admitted to the ACH program between July 6, 2020, and March 31, 2022. A total of 11 patients were excluded due to missing or unknown data, so 923 patients were analyzed. Demographic characteristics showed a mean age of 70.88 (SD 14.46), 54.3% were male, and the average LOS was 4.10 days. The 30-day readmission rate after discharge from ACH was 11.2% (*n* = 103) and the 30-day mortality rate was 1.8% (*n* = 17). In addition, the average SOI was 2.89 (SD 0.81), and the ROM was 2.73 (SD 0.92) which stands in a range between “Moderate” and “Major” in the APR-DRG classification (Table 1). There were no ACH inpatient mortalities recorded during the study period. The most frequent diagnosis was COVID-19 infection with 162 patients (17.6%), followed by heart failure exacerbation (12.7%) and septicemia (10.9%). Infectious diagnosis made up 53.6% of cases (*n* = 495), followed by cardiovascular disease (14.2%), surgical diagnosis (6.8%), airway diseases (5.5%), gastrointestinal / hepatobiliary disease (5.5%), kidney and urologic disease (5.2%), hematologic and oncologic disease (2.6%), musculoskeletal disease (2.1%), and endocrine disease (2.1%) (Table 2). Patients in the “Major” classification were the most frequent among all locations with a total of 378 (ROM) and 459 (SOI) patients out of the 923 (Table 3).

**Table 1:** Patient Demographics, Average Length of Stay, 30-Day Readmission Rate, 30-Day Mortality Rate, Severity of Illness, and Risk of Mortality of All ACH Patients

	**MCF**	**MCA**	**NWWI**	**TOTAL**
Patients	543	43	337	923
Mean Age (years)	70.88 (SD 14.46)	71.82 (SD 14.37)	70.90 (SD 14.48)	70.88 (SD 14.46)
Male patients (%)	53.2% (*n* = 289)	55.8% (*n* = 24)	55.7% (*n* = 188)	54.3% (*n* = 501)
Avg. LOS (days)	4.09	4.25	4.10	4.10
30-day Readmission Rate (%)	11.6% (*n* = 63)	4.7% (*n* = 2)	11.3% (*n* = 38)	11.2% (*n* = 103)
30-day Mortality Rate (%)	1.7% (*n* = 9)	0% (*n* = 0)	2.4% (*n* = 8)	1.8% (*n* = 17)
Severity of Illness (Avg.)	2.98 (SD 0.78)	3.02 (SD 0.82)	2.72 (SD 0.82)	2.89 (SD 0.81)
Risk of Mortality (Avg.)	2.81 (SD 0.92)	3.02 (SD 0.85)	2.58 (SD 0.91)	2.73 (SD 0.92)

*Abbreviations: ACH* Advance care at home, *MCF* Mayo Clinic Florida, *MCA* Mayo Clinic Arizona, *NWWI* Northwest Wisconsin, *SD* Standard deviation, *LOS* Length of stay, *Avg* Average

**Table 2:** Patient Diagnosis

	**Total** ***n* = 923**
**Infection**	495 (53.6%)
COVID-19 / COVID Pneumonia	162 (17.6%)
All other Pneumonia	65 (7.0%)
Septicemia / Bacteremia Complicated	101 (10.9%)
Skin / Soft Tissue infection	67 (7.3%)
UTI or pyelonephritis	45 (4.9%)
Infective Arthritis or Osteomyelitis	21 (2.3%)
Gastroenteritis / Intestinal Infection	14 (1.5%)
Peritonitis and intra-abdominal abscess	13 (1.4%)
Other infection	7 (0.8%)
**Cardiovascular disease**	131 (14.2%)
Heart Failure	117 (12.7%)
Cardiac Dysrhythmia	6 (0.7%)
Circulatory disease	4 (0.4%)
Other Cardiac	4 (0.4%)
**Hemotologic and oncologic disease**	24 (2.6%)
Cancer / Neoplastic	13 (1.4%)
Venous Thromboembolism	5 (0.5%)
Anemia / Neutropenia	6 (0.7%)
**Airway disease**	51 (5.5%)
COPD	21 (2.3%)
Aspiration pneumonitis	10 (1.1%)
Respiratory failure	9 (1.0%)
Bronchitis or sinusitis	4 (0.4%)
Other Airway Disease	7 (0.8%)
**Surgical diagnosis**	63 (6.8%)
Complication after surgery	29 (3.1%)
Complication of a device, implant, or graft	20 (2.2%)
Complication of Transplanted Tissue	14 (1.5%)
**Gastrointestinal and hepatobiliary disease**	51 (5.5%)
Biliary tract disease	14 (1.5%)
Diverticulosis and diverticulitis	8 (0.9%)
Oral and esophageal	8 (0.9%)
Pancreatitis	5 (0.5%)
Intestinal obstruction or ileus	3 (0.3%)
Inflammatory Bowel Disease	2 (0.2%)
Other gastrointestinal and hepatobiliary	11 (1.2%)
**Kidney and urologic disease**	48 (5.2%)
Acute renal failure	28 (3.0%)
Chronic kidney disease complication	7 (0.8%)
Fluid and electrolyte disorders	7 (0.8%)
Other Kidney and Urologic	6 (0.7%)
**Musculoskeletal disease**	19 (2.1%)
Osteoarthritis	7 (0.8%)
Pressure ulcer of skin	5 (0.5%)
Other Musculoskeletal	7 (0.8%)
**Endocrine disease**	19 (2.1%)
Diabetes mellitus with complication	15 (1.6%)
Pituitary disorders	4 (0.4%)
**Other / miscellaneous**	22 (2.4%)

**Table 3:** Frequencies of APR-DRG ROM and SOI Classification by Location

***For All ACH Patients (n = 923)***								
	**MCF**		**MCA**		**NWWI**		**TOTAL**	
	**ROM**	**SOI**	**ROM**	**SOI**	**ROM**	**SOI**	**ROM**	**SOI**
**Minor (1)**	51	17	3	2	46	22	100	41
**Moderate (2)**	138	121	6	8	101	88	245	217
**Major (3)**	218	260	21	20	139	179	378	459
**Extreme (4)**	136	145	13	13	51	48	200	206
***Excluding COVID-19 patients (n = 761)***								
	**MCF**		**MCA**		**NWWI**		**TOTAL**	
	**ROM**	**SOI**	**ROM**	**SOI**	**ROM**	**SOI**	**ROM**	**SOI**
**Minor (1)**	51	17	3	2	46	22	100	41
**Moderate (2)**	130	121	6	8	91	87	227	216
**Major (3)**	184	217	17	16	107	133	308	366
**Extreme (4)**	88	98	6	6	32	34	126	138

*Abbreviations: MCF* Mayo Clinic Florida, *MCA* Mayo Clinic Arizona, *NWWI* Northwest Wisconsin, *ROM* Risk of mortality, *SOI* Severity of illness

As the COVID-19 pandemic began and extended through our data collection and since COVID-19 was our largest cohort of diagnosis seen (*n*= 162), we decided to run a second analysis excluding these patients to determine if the SOI and ROM were inflated in value due to the number of COVID-19 patients, as it has been reported that hospitalized COVID-19 patients have higher ROM and SOI [13]. With the COVID-19 patients excluded, the mean patient age was 72.02 (SD 14.00), 53.9% were male, ALOS was 4.31, SOI was 2.79 (SD 0.80) and the ROM was 2.61 (SD 0.91) (Tables 3 and 4). The COVID-19 cohort (*n* = 162) had a SOI of 3.41 and a ROM of 3.35, which was significantly higher than the non-COVID-19 cohort (*p* < 0.001) (Table 5).

**Table 4:** Patient Demographics, Average Length of Stay, Severity of Illness, and Risk of Mortality Excluding COVID-19 Patients

	**MCF**	**MCA**	**NWWI**	**Total**
Patients	453	32	276	761
Mean Age (years)	72.00 (SD 14.63)	74.12 (SD 9.08)	71.80 (SD 13.40)	72.02 (SD 14.00)
Male patients (%)	52.31% (*n* = 237)	65.62% (*n* = 21)	55.07% (*n* = 152)	53.88% (*n* = 410)
Avg. LOS (days)	4.39	3.78	4.24	4.31
Severity of Illness (Avg.)	2.87 (SD 0.79)	2.81 (SD 0.81)	2.65 (SD 0.80)	2.79 (SD 0.80)
Risk of Mortality (Avg.)	2.68 (SD 0.91)	2.81 (SD 0.85)	2.46 (SD 0.91)	2.61 (SD 0.91)

*Abbreviations: ACH *Advance care at home, *MCF* Mayo Clinic Florida, *MCA* Mayo Clinic Arizona, *NWWI* Northwest Wisconsin, *SD* Standard deviation, *LOS* Length of stay, *Avg* Average

**Table 5:** Comparison of SOI and ROM between COVID-19 Patients and Non-COVID-19 Patients

	**COVID-19 Positive** **(*n* = 162)**	**COVID-19 Negative** **(*n* = 761)**	***P*-value**^**†**^
Severity of Illness (Avg.)	3.41	2.79	< 0.0001
Risk of Mortality (Avg.)	3.35	2.61	< 0.0001

*Abbreviations: ACH* Advance care at home, *MCF* Mayo Clinic Florida, *MCA* Mayo Clinic Arizona, *NWWI* Northwest Wisconsin, *SD* Standard deviation, *LOS* Length of stay, *Avg* Average

^**†**^Calculated by the unpaired Mann–Whitney U-test

## Discussion

To our knowledge, this is the first study to describe the acuity level of HaH patients in terms of APR-DRG SOI and ROM. We found that the overall average APR-DRG acuity level for ACH patients fell on the high end between “moderate” and “major”, with an average SOI of 2.89 and ROM of 2.73. This finding is crucially important as it illustrates the capability of ACH to care for very complex patients in the HaH virtual hybrid model. ACH patients are receiving high-acuity, inpatient level care lasting an average of about 4 days that would otherwise have to be performed within the walls of a physical hospital. This is a level of care that cannot be delivered in an acute care clinic or observational unit. Previous studies have looked at trying to address hospital capacity issues by setting up basic home services in order to move low acuity patients home [14, 15]. Our hybrid model of care [12] is able to combine virtual providers with an in-home medical support that can care for many different moderate to complex diagnosis as seen in Table 2. This model could be used by other institutions to address capacity issues by shifting moderately complex, clinically stable patients to an alternate setting for their care.

There are currently over 200 institutions that have applied for the Acute Hospital Care at Home waiver and are building their HaH programs [16, 17]. As these virtual and physical care models are built out, there is a need for standardization of acuity and capabilities of these many programs. Measuring the acuity of HaH patients with SOI and ROM is a reasonable way of evaluating the types of patients that programs are admitting. As we look at HaH programs’ emergency response teams, patient volume capacity, and resource needs, programs with higher APR-DRG acuity patients may need to be held to a higher standard in those areas in order to insure they can both provide the level of high-acuity care needed for their patients as well as respond promptly to any patient deteriorations. As we build better technologies and improve our HaH workflows, we must ensure that we never compromise patient safety or exceed the capacity capabilities of HaH [1]. Directing focus on patient acuity through APR-DRG SOI and ROM may help greatly in these efforts.

A last advantage of our findings is that they can be used as a tool to address concerns of both patients and clinicians who are not familiar or comfortable with the modern virtual hybrid HaH model of care. Skepticism in these programs still exists as many are still seen as low-acuity or glorified outpatient home care [14, 15]. Patients with complex medical disease may feel as if their acute care needs may not be met in HaH. Similarly, clinicians may be reluctant to recommend a program like ACH to their severely ill patients as they may feel that they are “too sick” for the program. Our findings of the high SOI and ROM in the majority of our ACH patients coupled with our in-program mortality rate of 0% should help reassure both patients and clinicians that a properly built HaH program can provide safe and effective inpatient care for high-acuity patients at home. This low mortality rate can mostly be attributed to the careful clinically screening process that is done prior to admitting a patient into ACH [12]. This clinical screening process (Additional file 1: Appendix A) detects patients that may either require higher acuity medical services to treat their primary diagnosis or who may be at high risk for rapid decompensation. For example, a COVID-19 patient could be moved into ACH for home treatment only if they met the following criteria: heart rate < 110 beats per minute, oxygen saturation level > 94% on 4 or less liters per minute of oxygen, and downtrending inflammatory markers on laboratory studies.

Many providers may have only recently had an experience with HaH or advanced home telemedicine in a single diagnosis group (COVID-19) during the recent pandemic. Some may be more comfortable with the capabilities of treating COVID-19 patients in this capacity but possibly not other diagnosis. Others might question if our findings were inflated by the known high SOI and ROM associated with the COVID-19 APR-DRG. Several of our findings in our second data analysis may address these concerns. First, we found that the average SOI and ROM of our COVID-19 patients (*n* = 162) was 3.41 and 3.35 respectively, supporting the belief that this cohort of patients is severely ill. Providers that did treat COVID-19 patients in HaH models may not have realized how severely ill they truly were, giving them more comfort to treat other high-acuity diagnosis at home. Second, we found that although the COVID-19 cohort had significantly higher SOI and ROM scores, even with this cohort of patients removed there was not a large decrease in our SOI or ROM (2.89 to 2.79 and 2.73 to 2.61 respectively), indicating that acuity of the non-COVID-19 patients still falls in the “moderate” to “major” category.

### Limitations

This study has several limitations. First of all, APR-DRG SOI and ROM are not comparable between diagnostic related groups, which means that a patient with a score of 4 or “extreme” in the cardiology diagnostic related groups is not comparable with a 4 in the neurology category. Additionally, potential causes of biased differences between expected and observed values derived from regression models can modify the result. These types of bias include having a disproportionate share of patients with the missing risk factor(s) or if the hospital consistently under or over reports risk factor(s) with influential effects on the model estimates and thus, alter the metric calculations. Lastly, if the statistical model is not properly calibrated and tested for systematic over or under estimation, then the model may systematically overestimate or underestimate expected values.

## Conclusion

The Advanced Care at Home virtual hybrid hospital-at-home program is capable of treating patients with “moderate” to “major” disease states, providing a realistic alternative for high-acuity care outside the brick-and-mortar hospital setting. Further investigations into patient acuity may both help build a more robust acceptance for ACH in patients with complex medical diseases as well as help standardize resource needs for patients in HaH programs with different acuity levels.

## Supplementary Information

**Additional file 1: Appendix A.** Clinical Stability Screening Tool.

## Data Availability

The Datasets used for this study are located on secured servers within Mayo Clinic that are only accessible by Mayo Clinic staff to protect any identified or deidentified patient information. Deidentified datasets used and/or analyzed during this study are available from the corresponding author on reasonable request.

## Abbreviations

ACH: Advanced Care at Home
HaH: Hospital-at-home
ED: Emergency Department
SOI: Severity of Illness
ROM: Risk of Mortality
APR-DRG: All Patient Refined-Diagnosis Related Groups
US: United States
CCI: Charlson comorbidity index
ALOS: Average Length of Stay
BAM: Brick-and-mortar
DRG: Diagnosis Related Groups
SD: Standard deviation
